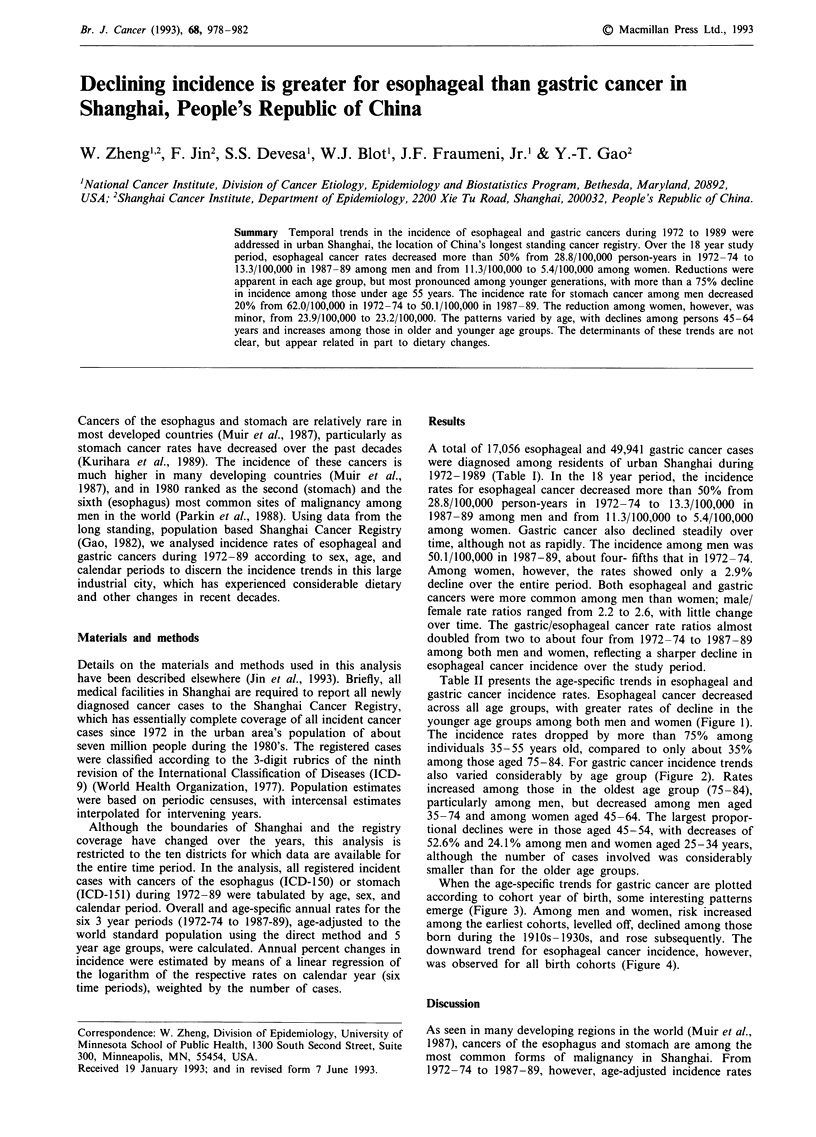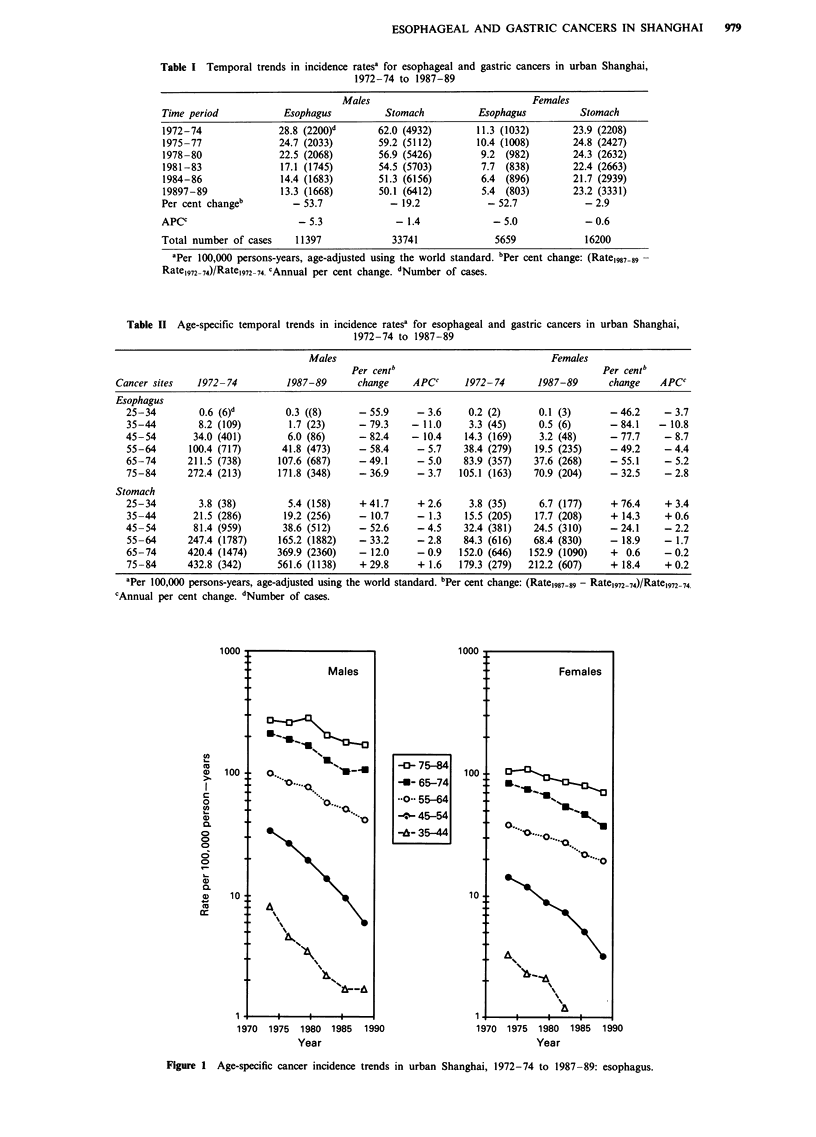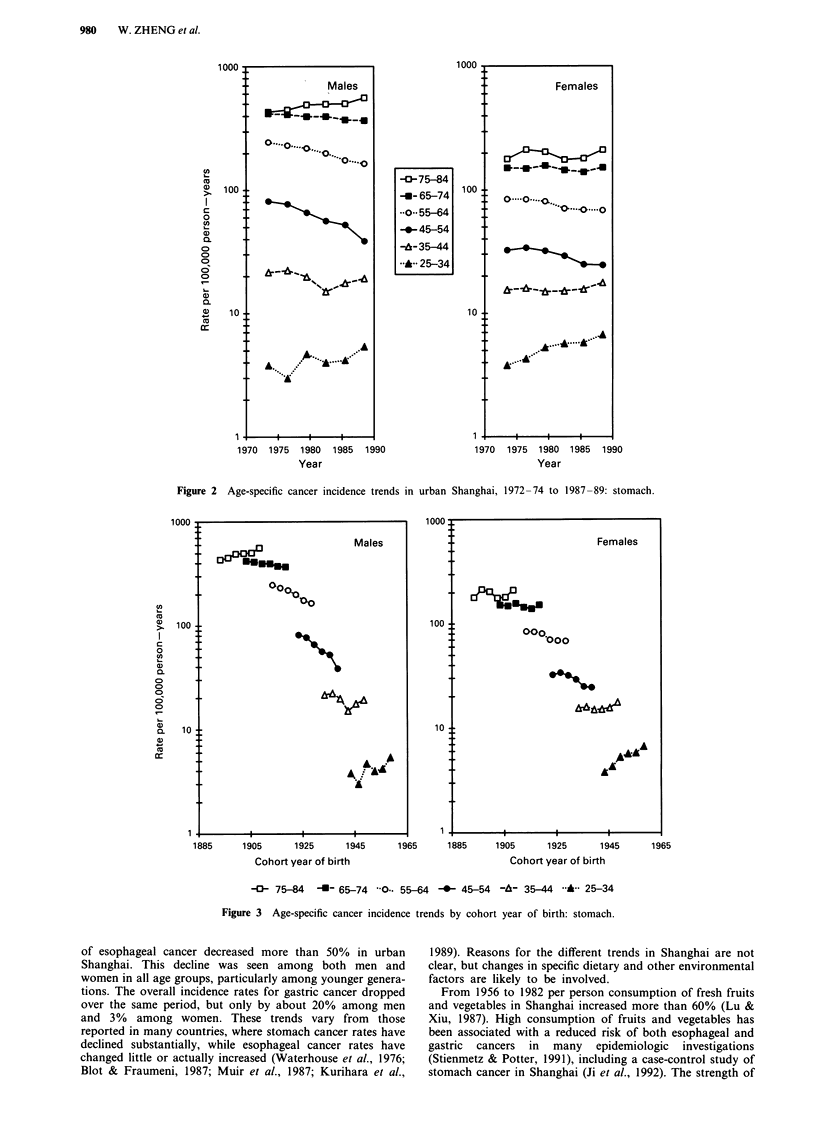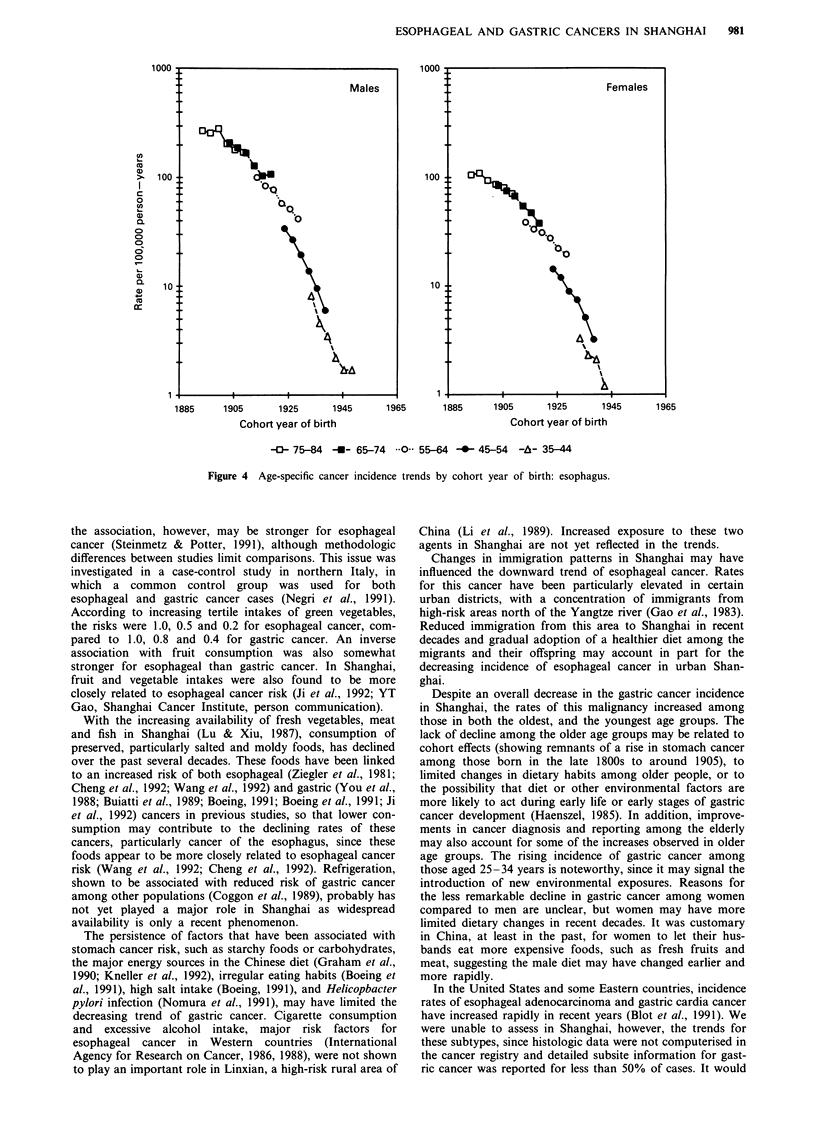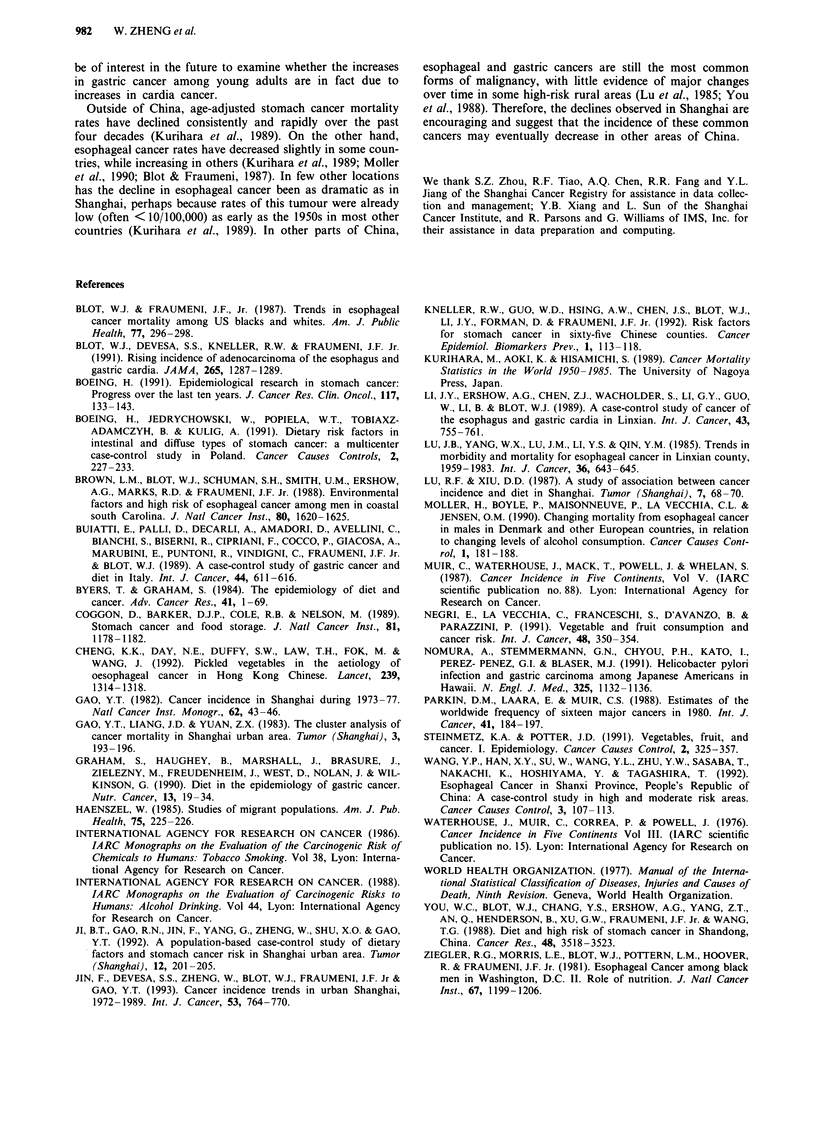# Declining incidence is greater for esophageal than gastric cancer in Shanghai, People's Republic of China.

**DOI:** 10.1038/bjc.1993.465

**Published:** 1993-11

**Authors:** W. Zheng, F. Jin, S. S. Devesa, W. J. Blot, J. F. Fraumeni, Y. T. Gao

**Affiliations:** National Cancer Institute, Division of Cancer Etiology, Bethesda, Maryland 20892.

## Abstract

Temporal trends in the incidence of esophageal and gastric cancers during 1972 to 1989 were addressed in urban Shanghai, the location of China's longest standing cancer registry. Over the 18 year study period, esophageal cancer rates decreased more than 50% from 28.8/100,000 person-years in 1972-74 to 13.3/100,000 in 1987-89 among men and from 11.3/100,000 to 5.4/100,000 among women. Reductions were apparent in each age group, but most pronounced among younger generations, with more than a 75% decline in incidence among those under age 55 years. The incidence rate for stomach cancer among men decreased 20% from 62.0/100,000 in 1972-74 to 50.1/100,000 in 1987-89. The reduction among women, however, was minor, from 23.9/100,000 to 23.2/100,000. The patterns varied by age, with declines among persons 45-64 years and increases among those in older and younger age groups. The determinants of these trends are not clear, but appear related in part to dietary changes.


Br. J. Cancer (1993), 68, 978-982 © Macmillan Press Ltd., 1993

### Declining incidence is greater for esophageal than gastric cancer in Shanghai, People's Republic of China

W. Zheng[1,2], F. Jin[2], S.S. Devesa[1], W.J. Blot[1], J.F. Fraumeni, Jr.[1] & Y.-T. Gao[2]

[1]*National Cancer Institute, Division of Cancer Etiology, Epidemiology and Biostatistics Program, Bethesda, Maryland, 20892, USA;* [2]*Shanghai Cancer Institute, Department of Epidemiology, 2200 Xie Tu Road, Shanghai, 200032, People's Republic of China.*

**Summary** Temporal trends in the incidence of esophageal and gastric cancers during 1972 to 1989 were addressed in urban Shanghai, the location of China's longest standing cancer registry. Over the 18 year study period, esophageal cancer rates decreased more than 50% from 28.8/100,000 person-years in 1972-74 to 13.3/100,000 in 1987-89 among men and from 11.3/100,000 to 5.4/100,000 among women. Reductions were apparent in each age group, but most pronounced among younger generations, with more than a 75% decline in incidence among those under age 55 years. The incidence rate for stomach cancer among men decreased 20% from 62.0/100,000 in 1972-74 to 50.1/100,000 in 1987-89. The reduction among women, however, was minor, from 23.9/100,000 to 23.2/100,000. The patterns varied by age, with declines among persons 45-64 years and increases among those in older and younger age groups. The determinants of these trends are not clear, but appear related in part to dietary changes.

Cancers of the esophagus and stomach are relatively rare in most developed countries (Muir *et al.*, 1987), particularly as stomach cancer rates have decreased over the past decades (Kurihara *et al.*, 1989). The incidence of these cancers is much higher in many developing countries (Muir *et al.*, 1987), and in 1980 ranked as the second (stomach) and the sixth (esophagus) most common sites of malignancy among men in the world (Parkin *et al.*, 1988). Using data from the long standing, population based Shanghai Cancer Registry (Gao, 1982), we analysed incidence rates of esophageal and gastric cancers during 1972-89 according to sex, age, and calendar periods to discern the incidence trends in this large industrial city, which has experienced considerable dietary and other changes in recent decades.

### Materials and methods

Details on the materials and methods used in this analysis have been described elsewhere (Jin *et al.*, 1993). Briefly, all medical facilities in Shanghai are required to report all newly diagnosed cancer cases to the Shanghai Cancer Registry, which has essentially complete coverage of all incident cancer cases since 1972 in the urban area's population of about seven million people during the 1980's. The registered cases were classified according to the 3-digit rubrics of the ninth revision of the International Classification of Diseases (ICD-9) (World Health Organization, 1977). Population estimates were based on periodic censuses, with intercensal estimates interpolated for intervening years.

Although the boundaries of Shanghai and the registry coverage have changed over the years, this analysis is restricted to the ten districts for which data are available for the entire time period. In the analysis, all registered incident cases with cancers of the esophagus (ICD-150) or stomach (ICD-151) during 1972-89 were tabulated by age, sex, and calendar period. Overall and age-specific annual rates for the six 3 year periods (1972-74 to 1987-89), age-adjusted to the world standard population using the direct method and 5 year age groups, were calculated. Annual percent changes in incidence were estimated by means of a linear regression of the logarithm of the respective rates on calendar year (six time periods), weighted by the number of cases.

Correspondence: W. Zheng, Division of Epidemiology, University of Minnesota School of Public Health, 1300 South Second Street, Suite 300, Minneapolis, MN, 55454, USA.
Received 19 January 1993; and in revised form 7 June 1993.

### Results

A total of 17,056 esophageal and 49,941 gastric cancer cases were diagnosed among residents of urban Shanghai during 1972-1989 (Table I). In the 18 year period, the incidence rates for esophageal cancer decreased more than 50% from 28.8/100,000 person-years in 1972-74 to 13.3/100,000 in 1987-89 among men and from 11.3/100,000 to 5.4/100,000 among women. Gastric cancer also declined steadily over time, although not as rapidly. The incidence among men was 50.1/100,000 in 1987-89, about four- fifths that in 1972-74. Among women, however, the rates showed only a 2.9% decline over the entire period. Both esophageal and gastric cancers were more common among men than women; male/female rate ratios ranged from 2.2 to 2.6, with little change over time. The gastric/esophageal cancer rate ratios almost doubled from two to about four from 1972-74 to 1987-89 among both men and women, reflecting a sharper decline in esophageal cancer incidence over the study period.

Table II presents the age-specific trends in esophageal and gastric cancer incidence rates. Esophageal cancer decreased across all age groups, with greater rates of decline in the younger age groups among both men and women (Figure 1). The incidence rates dropped by more than 75% among individuals 35-55 years old, compared to only about 35% among those aged 75-84. For gastric cancer incidence trends also varied considerably by age group (Figure 2). Rates increased among those in the oldest age group (75-84), particularly among men, but decreased among men aged 35-74 and among women aged 45-64. The largest proportional declines were in those aged 45-54, with decreases of 52.6% and 24.1% among men and women aged 25-34 years, although the number of cases involved was considerably smaller than for the older age groups.

When the age-specific trends for gastric cancer are plotted according to cohort year of birth, some interesting patterns emerge (Figure 3). Among men and women, risk increased among the earliest cohorts, levelled off, declined among those born during the 1910s-1930s, and rose subsequently. The downward trend for esophageal cancer incidence, however, was observed for all birth cohorts (Figure 4).

### Discussion

As seen in many developing regions in the world (Muir *et al.*, 1987), cancers of the esophagus and stomach are among the most common forms of malignancy in Shanghai. From 1972-74 to 1987-89, however, age-adjusted incidence rates

**Table I** Temporal trends in incidence rates[a] for esophageal and gastric cancers in urban Shanghai, 1972–74 to 1987–89

| | *Males* | | *Females* | |
|---|---|---|---|---|
| *Time period* | *Esophagus* | *Stomach* | *Esophagus* | *Stomach* |
| 1972–74 | 28.8 (2200)[d] | 62.0 (4932) | 11.3 (1032) | 23.9 (2208) |
| 1975–77 | 24.7 (2033) | 59.2 (5112) | 10.4 (1008) | 24.8 (2427) |
| 1978–80 | 22.5 (2068) | 56.9 (5426) | 9.2 (982) | 24.3 (2632) |
| 1981–83 | 17.1 (1745) | 54.5 (5703) | 7.7 (838) | 22.4 (2663) |
| 1984–86 | 14.4 (1683) | 51.3 (6156) | 6.4 (896) | 21.7 (2939) |
| 19897–89 | 13.3 (1668) | 50.1 (6412) | 5.4 (803) | 23.2 (3331) |
| Per cent change[b] | – 53.7 | – 19.2 | – 52.7 | – 2.9 |
| APC[c] | – 5.3 | – 1.4 | – 5.0 | – 0.6 |
| Total number of cases | 11397 | 33741 | 5659 | 16200 |

[a]Per 100,000 persons-years, age-adjusted using the world standard. [b]Per cent change: $(Rate_{1987-89} - Rate_{1972-74})/Rate_{1972-74}$. [c]Annual per cent change. [d]Number of cases.

**Table II** Age-specific temporal trends in incidence rates[a] for esophageal and gastric cancers in urban Shanghai, 1972–74 to 1987–89

| | *Males* | | | | *Females* | | | |
|---|---|---|---|---|---|---|---|---|
| *Cancer sites* | *1972–74* | *1987–89* | *Per cent[b] change* | *APC[c]* | *1972–74* | *1987–89* | *Per cent[b] change* | *APC[c]* |
| *Esophagus* | | | | | | | | |
| 25–34 | 0.6 (6)[d] | 0.3 ((8) | – 55.9 | – 3.6 | 0.2 (2) | 0.1 (3) | – 46.2 | – 3.7 |
| 35–44 | 8.2 (109) | 1.7 (23) | – 79.3 | – 11.0 | 3.3 (45) | 0.5 (6) | – 84.1 | – 10.8 |
| 45–54 | 34.0 (401) | 6.0 (86) | – 82.4 | – 10.4 | 14.3 (169) | 3.2 (48) | – 77.7 | – 8.7 |
| 55–64 | 100.4 (717) | 41.8 (473) | – 58.4 | – 5.7 | 38.4 (279) | 19.5 (235) | – 49.2 | – 4.4 |
| 65–74 | 211.5 (738) | 107.6 (687) | – 49.1 | – 5.0 | 83.9 (357) | 37.6 (268) | – 55.1 | – 5.2 |
| 75–84 | 272.4 (213) | 171.8 (348) | – 36.9 | – 3.7 | 105.1 (163) | 70.9 (204) | – 32.5 | – 2.8 |
| *Stomach* | | | | | | | | |
| 25–34 | 3.8 (38) | 5.4 (158) | + 41.7 | + 2.6 | 3.8 (35) | 6.7 (177) | + 76.4 | + 3.4 |
| 35–44 | 21.5 (286) | 19.2 (256) | – 10.7 | – 1.3 | 15.5 (205) | 17.7 (208) | + 14.3 | + 0.6 |
| 45–54 | 81.4 (959) | 38.6 (512) | – 52.6 | – 4.5 | 32.4 (381) | 24.5 (310) | – 24.1 | – 2.2 |
| 55–64 | 247.4 (1787) | 165.2 (1882) | – 33.2 | – 2.8 | 84.3 (616) | 68.4 (830) | – 18.9 | – 1.7 |
| 65–74 | 420.4 (1474) | 369.9 (2360) | – 12.0 | – 0.9 | 152.0 (646) | 152.9 (1090) | + 0.6 | – 0.2 |
| 75–84 | 432.8 (342) | 561.6 (1138) | + 29.8 | + 1.6 | 179.3 (279) | 212.2 (607) | + 18.4 | + 0.2 |

[a]Per 100,000 persons-years, age-adjusted using the world standard. [b]Per cent change: $(Rate_{1987-89} - Rate_{1972-74})/Rate_{1972-74}$. [c]Annual per cent change. [d]Number of cases.

**Figure 1** Age-specific cancer incidence trends in urban Shanghai, 1972–74 to 1987–89: esophagus.

**Figure 2** Age-specific cancer incidence trends in urban Shanghai, 1972–74 to 1987–89: stomach.

**Figure 3** Age-specific cancer incidence trends by cohort year of birth: stomach.

of esophageal cancer decreased more than 50% in urban Shanghai. This decline was seen among both men and women in all age groups, particularly among younger generations. The overall incidence rates for gastric cancer dropped over the same period, but only by about 20% among men and 3% among women. These trends vary from those reported in many countries, where stomach cancer rates have declined substantially, while esophageal cancer rates have changed little or actually increased (Waterhouse *et al.*, 1976; Blot & Fraumeni, 1987; Muir *et al.*, 1987; Kurihara *et al.*, 1989). Reasons for the different trends in Shanghai are not clear, but changes in specific dietary and other environmental factors are likely to be involved.

From 1956 to 1982 per person consumption of fresh fruits and vegetables in Shanghai increased more than 60% (Lu & Xiu, 1987). High consumption of fruits and vegetables has been associated with a reduced risk of both esophageal and gastric cancers in many epidemiologic investigations (Stienmetz & Potter, 1991), including a case-control study of stomach cancer in Shanghai (Ji *et al.*, 1992). The strength of

**Figure 4** Age-specific cancer incidence trends by cohort year of birth: esophagus.

the association, however, may be stronger for esophageal cancer (Steinmetz & Potter, 1991), although methodologic differences between studies limit comparisons. This issue was investigated in a case-control study in northern Italy, in which a common control group was used for both esophageal and gastric cancer cases (Negri *et al.*, 1991). According to increasing tertile intakes of green vegetables, the risks were 1.0, 0.5 and 0.2 for esophageal cancer, compared to 1.0, 0.8 and 0.4 for gastric cancer. An inverse association with fruit consumption was also somewhat stronger for esophageal than gastric cancer. In Shanghai, fruit and vegetable intakes were also found to be more closely related to esophageal cancer risk (Ji *et al.*, 1992; YT Gao, Shanghai Cancer Institute, person communication).

With the increasing availability of fresh vegetables, meat and fish in Shanghai (Lu & Xiu, 1987), consumption of preserved, particularly salted and moldy foods, has declined over the past several decades. These foods have been linked to an increased risk of both esophageal (Ziegler *et al.*, 1981; Cheng *et al.*, 1992; Wang *et al.*, 1992) and gastric (You *et al.*, 1988; Buiatti *et al.*, 1989; Boeing, 1991; Boeing *et al.*, 1991; Ji *et al.*, 1992) cancers in previous studies, so that lower consumption may contribute to the declining rates of these cancers, particularly cancer of the esophagus, since these foods appear to be more closely related to esophageal cancer risk (Wang *et al.*, 1992; Cheng *et al.*, 1992). Refrigeration, shown to be associated with reduced risk of gastric cancer among other populations (Coggon *et al.*, 1989), probably has not yet played a major role in Shanghai as widespread availability is only a recent phenomenon.

The persistence of factors that have been associated with stomach cancer risk, such as starchy foods or carbohydrates, the major energy sources in the Chinese diet (Graham *et al.*, 1990; Kneller *et al.*, 1992), irregular eating habits (Boeing *et al.*, 1991), high salt intake (Boeing, 1991), and *Helicopbacter pylori* infection (Nomura *et al.*, 1991), may have limited the decreasing trend of gastric cancer. Cigarette consumption and excessive alcohol intake, major risk factors for esophageal cancer in Western countries (International Agency for Research on Cancer, 1986, 1988), were not shown to play an important role in Linxian, a high-risk rural area of China (Li *et al.*, 1989). Increased exposure to these two agents in Shanghai are not yet reflected in the trends.

Changes in immigration patterns in Shanghai may have influenced the downward trend of esophageal cancer. Rates for this cancer have been particularly elevated in certain urban districts, with a concentration of immigrants from high-risk areas north of the Yangtze river (Gao *et al.*, 1983). Reduced immigration from this area to Shanghai in recent decades and gradual adoption of a healthier diet among the migrants and their offspring may account in part for the decreasing incidence of esophageal cancer in urban Shanghai.

Despite an overall decrease in the gastric cancer incidence in Shanghai, the rates of this malignancy increased among those in both the oldest, and the youngest age groups. The lack of decline among the older age groups may be related to cohort effects (showing remnants of a rise in stomach cancer among those born in the late 1800s to around 1905), to limited changes in dietary habits among older people, or to the possibility that diet or other environmental factors are more likely to act during early life or early stages of gastric cancer development (Haenszel, 1985). In addition, improvements in cancer diagnosis and reporting among the elderly may also account for some of the increases observed in older age groups. The rising incidence of gastric cancer among those aged 25–34 years is noteworthy, since it may signal the introduction of new environmental exposures. Reasons for the less remarkable decline in gastric cancer among women compared to men are unclear, but women may have more limited dietary changes in recent decades. It was customary in China, at least in the past, for women to let their husbands eat more expensive foods, such as fresh fruits and meat, suggesting the male diet may have changed earlier and more rapidly.

In the United States and some Eastern countries, incidence rates of esophageal adenocarcinoma and gastric cardia cancer have increased rapidly in recent years (Blot *et al.*, 1991). We were unable to assess in Shanghai, however, the trends for these subtypes, since histologic data were not computerised in the cancer registry and detailed subsite information for gastric cancer was reported for less than 50% of cases. It would be of interest in the future to examine whether the increases in gastric cancer among young adults are in fact due to increases in cardia cancer.

Outside of China, age-adjusted stomach cancer mortality rates have declined consistently and rapidly over the past four decades (Kurihara *et al*., 1989). On the other hand, esophageal cancer rates have decreased slightly in some countries, while increasing in others (Kurihara *et al*., 1989; Moller *et al*., 1990; Blot & Fraumeni, 1987). In few other locations has the decline in esophageal cancer been as dramatic as in Shanghai, perhaps because rates of this tumour were already low (often $<10/100,000$) as early as the 1950s in most other countries (Kurihara *et al*., 1989). In other parts of China, esophageal and gastric cancers are still the most common forms of malignancy, with little evidence of major changes over time in some high-risk rural areas (Lu *et al*., 1985; You *et al*., 1988). Therefore, the declines observed in Shanghai are encouraging and suggest that the incidence of these common cancers may eventually decrease in other areas of China.

We thank S.Z. Zhou, R.F. Tiao, A.Q. Chen, R.R. Fang and Y.L. Jiang of the Shanghai Cancer Registry for assistance in data collection and management; Y.B. Xiang and L. Sun of the Shanghai Cancer Institute, and R. Parsons and G. Williams of IMS, Inc. for their assistance in data preparation and computing.

### References

BLOT, W.J. & FRAUMENI, J.F., Jr. (1987). Trends in esophageal cancer mortality among US blacks and whites. *Am. J. Public Health*, **77**, 296–298.

BLOT, W.J., DEVESA, S.S., KNELLER, R.W. & FRAUMENI, J.F. Jr. (1991). Rising incidence of adenocarcinoma of the esophagus and gastric cardia. *JAMA*, **265**, 1287–1289.

BOEING, H. (1991). Epidemiological research in stomach cancer: Progress over the last ten years. *J. Cancer Res. Clin. Oncol.*, **117**, 133–143.

BOEING, H., JEDRYCHOWSKI, W., POPIELA, W.T., TOBIAXZ-ADAMCZYH, B. & KULIG, A. (1991). Dietary risk factors in intestinal and diffuse types of stomach cancer: a multicenter case-control study in Poland. *Cancer Causes Controls*, **2**, 227–233.

BROWN, L.M., BLOT, W.J., SCHUMAN, S.H., SMITH, U.M., ERSHOW, A.G., MARKS, R.D. & FRAUMENI, J.F. Jr. (1988). Environmental factors and high risk of esophageal cancer among men in coastal south Carolina. *J. Natl Cancer Inst.*, **80**, 1620–1625.

BUIATTI, E., PALLI, D., DECARLI, A., AMADORI, D., AVELLINI, C., BIANCHI, S., BISERNI, R., CIPRIANI, F., COCCO, P., GIACOSA, A., MARUBINI, E., PUNTONI, R., VINDIGNI, C., FRAUMENI, J.F. Jr. & BLOT, W.J. (1989). A case-control study of gastric cancer and diet in Italy. *Int. J. Cancer*, **44**, 611–616.

BYERS, T. & GRAHAM, S. (1984). The epidemiology of diet and cancer. *Adv. Cancer Res.*, **41**, 1–69.

COGGON, D., BARKER, D.J.P., COLE, R.B. & NELSON, M. (1989). Stomach cancer and food storage. *J. Natl Cancer Inst.*, **81**, 1178–1182.

CHENG, K.K., DAY, N.E., DUFFY, S.W., LAW, T.H., FOK, M. & WANG, J. (1992). Pickled vegetables in the aetiology of oesophageal cancer in Hong Kong Chinese. *Lancet*, **239**, 1314–1318.

GAO, Y.T. (1982). Cancer incidence in Shanghai during 1973–77. *Natl Cancer Inst. Monogr.*, **62**, 43–46.

GAO, Y.T., LIANG, J.D. & YUAN, Z.X. (1983). The cluster analysis of cancer mortality in Shanghai urban area. *Tumor (Shanghai)*, **3**, 193–196.

GRAHAM, S., HAUGHEY, B., MARSHALL, J., BRASURE, J., ZIELEZNY, M., FREUDENHEIM, J., WEST, D., NOLAN, J. & WILKINSON, G. (1990). Diet in the epidemiology of gastric cancer. *Nutr. Cancer*, **13**, 19–34.

HAENSZEL, W. (1985). Studies of migrant populations. *Am. J. Pub. Health*, **75**, 225–226.

INTERNATIONAL AGENCY FOR RESEARCH ON CANCER (1986). *IARC Monographs on the Evaluation of the Carcinogenic Risk of Chemicals to Humans: Tobacco Smoking*. Vol 38, Lyon: International Agency for Research on Cancer.

INTERNATIONAL AGENCY FOR RESEARCH ON CANCER. (1988). *IARC Monographs on the Evaluation of Carcinogenic Risks to Humans: Alcohol Drinking*. Vol 44, Lyon: International Agency for Research on Cancer.

JI, B.T., GAO, R.N., JIN, F., YANG, G., ZHENG, W., SHU, X.O. & GAO, Y.T. (1992). A population-based case-control study of dietary factors and stomach cancer risk in Shanghai urban area. *Tumor (Shanghai)*, **12**, 201–205.

JIN, F., DEVESA, S.S., ZHENG, W., BLOT, W.J., FRAUMENI, J.F Jr & GAO, Y.T. (1993). Cancer incidence trends in urban Shanghai, 1972–1989. *Int. J. Cancer*, **53**, 764–770.

KNELLER, R.W., GUO, W.D., HSING, A.W., CHEN, J.S., BLOT, W.J., LI, J.Y., FORMAN, D. & FRAUMENI, J.F. Jr. (1992). Risk factors for stomach cancer in sixty-five Chinese counties. *Cancer Epidemiol. Biomarkers Prev.*, **1**, 113–118.

KURIHARA, M., AOKI, K. & HISAMICHI, S. (1989). *Cancer Mortality Statistics in the World 1950–1985*. The University of Nagoya Press, Japan.

LI, J.Y., ERSHOW, A.G., CHEN, Z.J., WACHOLDER, S., LI, G.Y., GUO, W., LI, B. & BLOT, W.J. (1989). A case-control study of cancer of the esophagus and gastric cardia in Linxian. *Int. J. Cancer*, **43**, 755–761.

LU, J.B., YANG, W.X., LU, J.M., LI, Y.S. & QIN, Y.M. (1985). Trends in morbidity and mortality for esophageal cancer in Linxian county, 1959–1983. *Int. J. Cancer*, **36**, 643–645.

LU, R.F. & XIU, D.D. (1987). A study of association between cancer incidence and diet in Shanghai. *Tumor (Shanghai)*, **7**, 68–70.

MOLLER, H., BOYLE, P., MAISONNEUVE, P., LA VECCHIA, C.L. & JENSEN, O.M. (1990). Changing mortality from esophageal cancer in males in Denmark and other European countries, in relation to changing levels of alcohol consumption. *Cancer Causes Control*, **1**, 181–188.

MUIR, C., WATERHOUSE, J., MACK, T., POWELL, J. & WHELAN, S. (1987). *Cancer Incidence in Five Continents*, Vol V. (IARC scientific publication no. 88). Lyon: International Agency for Research on Cancer.

NEGRI, E., LA VECCHIA, C., FRANCESCHI, S., D'AVANZO, B. & PARAZZINI, P. (1991). Vegetable and fruit consumption and cancer risk. *Int. J. Cancer*, **48**, 350–354.

NOMURA, A., STEMMERMANN, G.N., CHYOU, P.H., KATO, I., PEREZ- PENEZ, G.I. & BLASER, M.J. (1991). Helicobacter pylori infection and gastric carcinoma among Japanese Americans in Hawaii. *N. Engl. J. Med.*, **325**, 1132–1136.

PARKIN, D.M., LAARA, E. & MUIR, C.S. (1988). Estimates of the worldwide frequency of sixteen major cancers in 1980. *Int. J. Cancer*, **41**, 184–197.

STEINMETZ, K.A. & POTTER, J.D. (1991). Vegetables, fruit, and cancer. I. Epidemiology. *Cancer Causes Control*, **2**, 325–357.

WANG, Y.P., HAN, X.Y., SU, W., WANG, Y.L., ZHU, Y.W., SASABA, T., NAKACHI, K., HOSHIYAMA, Y. & TAGASHIRA, T. (1992). Esophageal Cancer in Shanxi Province, People's Republic of China: A case-control study in high and moderate risk areas. *Cancer Causes Control*, **3**, 107–113.

WATERHOUSE, J., MUIR, C., CORREA, P. & POWELL, J. (1976). *Cancer Incidence in Five Continents* Vol III. (IARC scientific publication no. 15). Lyon: International Agency for Research on Cancer.

WORLD HEALTH ORGANIZATION. (1977). *Manual of the International Statistical Classification of Diseases, Injuries and Causes of Death, Ninth Revision*. Geneva, World Health Organization.

YOU, W.C., BLOT, W.J., CHANG, Y.S., ERSHOW, A.G., YANG, Z.T., AN, Q., HENDERSON, B., XU, G.W., FRAUMENI, J.F. Jr. & WANG, T.G. (1988). Diet and high risk of stomach cancer in Shandong, China. *Cancer Res.*, **48**, 3518–3523.

ZIEGLER, R.G., MORRIS, L.E., BLOT, W.J., POTTERN, L.M., HOOVER, R. & FRAUMENI, J.F. Jr. (1981). Esophageal Cancer among black men in Washington, D.C. II. Role of nutrition. *J. Natl Cancer Inst.*, **67**, 1199–1206.